# Memory Recovery Effect of a New Bioactive Innovative Combination in Rats with Experimental Dementia

**DOI:** 10.3390/antiox12122050

**Published:** 2023-11-28

**Authors:** Lyubka Tancheva, Reni Kalfin, Borislav Minchev, Diamara Uzunova, Krasimira Tasheva, Elina Tsvetanova, Almira Georgieva, Albena Alexandrova, Miroslava Stefanova, Ayten Solak, Maria Lazarova, Yordan Hodzhev, Valya Grigorova, Dobri Yarkov, Polina Petkova-Kirova

**Affiliations:** 1 Institute of Neurobiology, Bulgarian Academy of Sciences, Acad. G. Bonchev Str. 23, 1113 Sofia, Bulgaria; l.tancheva@inb.bas.bg (L.T.); boby_al@mail.bg (B.M.); didi_uzunova1@abv.bg (D.U.); elina_nesta@abv.bg (E.T.); a.georgieva@inb.bas.bg (A.G.); a.alexandrova@inb.bas.bg (A.A.); mira_stefanova@mail.bg (M.S.); asolak@abv.bg (A.S.); m.lazarova@gmail.com (M.L.);; 2 Department of Healthcare, South-West University “Neofit Rilski”, Ivan Mihailov Str. 66, 2700 Blagoevgrad, Bulgaria; 3 Institute of Plant Physiology and Genetics, Bulgarian Academy of Sciences, Acad. G. Bonchev Str. 21, 1113 Sofia, Bulgaria; krasitasheva@abv.bg; 4 National Sports Academy, Department of Physiology and Biochemistry, Acad. S. Mladenov Str. 21, 1700 Sofia, Bulgaria; 5 Institute of Cryobiology and Food Technologies, Cherni Vrah Blvd 53, 1407 Sofia, Bulgaria; 6 National Center of Infectious and Parasitic Diseases, Yanko Sakazov Blvd 26, 1504 Sofia, Bulgaria; jordanqvo@gmail.com; 7 Faculty of Veterinary Medicine, Trakia University, 6000 Stara Zagora, Bulgaria; rector@trakia-uni.bg

**Keywords:** Alzheimer’s disease, α-lipoic acid, citicoline, extract of leaves green tea, extract of leaves olive tree, vitamin D_3_, selenium, scopolamine

## Abstract

Alzheimer’s disease manifests as a complex pathological condition, with neuroinflammation, oxidative stress and cholinergic dysfunction being a few of the many pathological changes. Due to the complexity of the disease, current therapeutic strategies aim at a multitargeted approach, often relying on a combination of substances with versatile and complementary effects. In the present study, a unique combination of α-lipoic acid, citicoline, extracts of leaves from olive tree and green tea, vitamin D3, selenium and an immune-supporting complex was tested in scopolamine-induced dementia in rats. Using behavioral and biochemical methods, we assessed the effects of the combination on learning and memory, and elucidated the mechanisms of these effects. Our results showed that, compared to its components, the experimental combination was most efficient in improving short- and long-term memory as assessed by the step-through method as well as spatial memory as assessed by T-maze and Barnes maze underlined by decreases in AChE activity (*p* < 0.05) and LPO (*p* < 0.001), increases in SOD activity in the cortex (*p* < 0.05) and increases in catalase (*p* < 0.05) and GPx (*p* < 0.01) activities and BDNF (*p* < 0.001) and pCREB (*p* < 0.05) levels in the hippocampus. No significant histopathological changes or blood parameter changes were detected, making the experimental combination an effective and safe candidate in a multitargeted treatment of AD.

## 1. Introduction

Alzheimer’s disease (AD), the most common form of dementia (contributing to 60–70% of cases), is often referred to as the “epidemic of the 21st century”. According to the World Health Organization, at present, more than 55 million people suffer from dementia, the number increasing with 10 million new cases each year. AD and other forms of dementia are revealed as leading causes for death and disability, being classified among the top 10 causes of death worldwide and ranking 3rd in both the Americas and Europe in 2019 and described as one of the most important sources of incapacity and dependency among older people globally, turning the neurodegenerative condition into a major health care challenge.

Although deposition of extracellular senile plaques and the presence of intracellular neurofibrillary tangles are considered the major characteristics of AD, it manifests as a complex pathological condition, with neuroinflammation, oxidative stress, mitochondrial dysfunction and changes in the cholinergic and monoaminergic neurotransmitter systems being of significant importance [1,2,3,4,5,6,7,8,9]. Apoptosis, altered brain connectivity and aberrant neuronal activity as well as brain imbalance of metal ions such as iron, copper, zinc and calcium and impaired glucose metabolism are also shown to play an important role [10,11,12,13]. Thus, modern AD therapeutic strategies are increasingly aiming at applying a multitargeted approach to account for the large complexity of the neurodegenerative condition, the treatment and prevention of which so far remain ineffective. Due to their many-sided mechanisms of action and versatile beneficial effects for a range of diseases, biologically active substances of natural origin are coming steadily in the focus of attention of researchers and clinicians as possible treatment and preventive drugs. Many research groups, including ours, report on the favorable effects of natural substances and their synthetically modified analogues in experimental models of dementia and AD based on their antioxidative, anticholinesterase, anti-inflammatory, immunomodulatory and other properties [14,15,16,17,18,19,20,21,22,23,24,25,26,27,28,29,30].

Thus, relying on the combined effect and synergistic action of their components, pharmacological combinations based on naturally occurring substances can demonstrate improved effectiveness and turn promising tools as treatment and preventive AD drugs [31,32].

**Lipoic acid** (LA), also known as α-lipoic acid and thioctic acid, is a low molecular dithiol, naturally occurring in cells and functioning as an essential cofactor for mitochondrial enzymes [33]. It is found in both vegetable and animal-based food, most abundantly in spinach, broccolis, and tomatoes as well as bovine kidney, heart, and liver [34]. It affects multiple pathways and shows an abundance of effects that can improve cognitive performance, ensure and sustain neuroprotection and be considered in neurodegenerative disorders, especially AD. Lipoic acid is a powerful antioxidant as, in addition to reducing oxidative stress by scavenging reactive oxygen species (ROS), hypochlorous acid and NO and preventing oxidation of lipids and proteins, it regenerates other oxidants like vit C and E and increases the intracellular levels of glutathione (GSH) and ubiquinone [35,36,37]. As generally known, GSH is an essential key antioxidant with fundamental role in neutralizing ROS and regulating the intrinsic redox homeostasis.

Furthermore, LA and its reduced form dihydrolipoic acid (DHLA) chelate redox-active metals such as Co, Fe and Zn, the accumulation of which mediates free radical generation and oxidative damage related to neurodegeneration [33,38]. There is evidence that the iron-chelating ability decreases the phosphorylation of tau and the aggregation of Aβ monomers into harmful fibrous forms, thus delaying cell death [39]. Additionally, LA is effective against Aβ-induced cytotoxicity, most probably due to its free radical scavenging capacity as Aβ in senile plaques is demonstrated to stimulate production of free radicals [40].

LA possesses also anti-inflammatory properties once, by being a strong antioxidant, as elevated oxidative stress is related to chronic inflammation and second, by inhibiting the NF-κB signaling pathway which involves various genes expression implicated in cell apoptosis and inflammatory responses [41]. LA increases acetylcholine (ACh) production by activation of choline acetyltransferase and by increasing glucose uptake, thus supplying more acetyl-CoA for the production of ACh [42]. LA demonstrates antiapoptotic properties by inhibiting both caspase-dependent and caspase independent cell death pathways [43]. And last but not least, treatment with LA has been shown to increase the levels of neurotransmitters like dopamine, serotonin, and norepinephrine in various brain regions [44].

In view of the numerous favorable properties of LA, a wealth of evidence shows its ameliorating effects in cell and animal models of AD as well as when used by patients [27,45,46,47,48,49,50]. Several studies examine the outcome also from the use of combinations of LA with other natural substances like curcumin, piperine, epigallocatechin gallate, N-acetylcysteine, B vitamins, vitamin C and omega-3 fatty acids reporting positive effects on cognition and AD pathology both in an AD mouse model and AD patients [31,32,51]. Thus, LA poses itself as a serious candidate for taking a central part in a possible multitargeted AD therapy.

**Citicoline** (cytidine 5′-diphosphocholine), normally present in human cells, is an essential intermediate in the biosynthesis of cell membrane structural phospholipids, particularly, phosphatidylcholine, and a source of choline, used in the biosynthesis of acetylcholine [52]. Citicoline appears not less significant than LA as a possible AD treatment and preventive drug as it demonstrates an equally impressive array of physiological functions related to neuroprotective and procognitive effects. Apart from being involved in the biosynthesis of phosphatidylcholine, a primary component of neuronal membranes, and ACh, with a major role in memory and learning, it advances the synthesis of several other membrane phospholipids, including phosphatidylethanolamine and phosphatidylserine, contributing to neuronal repair and regeneration and acts as a selective agonist at nicotinic ACh receptors (choline at α7-nAChR), which have an important role in maintaining normal cognition. In cerebral ischemia and models of neurodegeneration, citicoline has been shown to inhibit apoptosis [52,53], to reduce hippocampal neuronal degeneration, to ameliorate learning and memory [53], to normalize norepinephrine and dopamine release [54], to inhibit phospholipase A2 (PLA2) thus preventing ROS and proinflammatory mediators generation from fatty acids, especially arachidonic acid [55]. Oxidative metabolism of arachidonic acid is considered a significant source of ROS [56]. Arachidonic acid is the direct precursor of bioactive lipid metabolites of eicosanoids such as prostaglandins, leukotrienes and epoxyeicosatrienoic acid, considered proinflammatory molecules, as they trigger oxidative stress and stimulate the immune response [57]. Moreover, citicoline stimulates glutathione synthesis and the activity of glutathione reductase, attenuates lipid peroxidation, facilitates mitochondrial function by increasing the activity of cytochrome oxidase [52] and restoring the activity of the mitochondrial ATPase [58,59] and levels of cardiolipin, a phospholipid indispensable for mitochondrial electron transport [52]. Actually, citicoline has been long recognized for its procognitive properties and is one of the most commonly prescribed drugs for cognitive impairments in several European countries [60]. Several recent reviews on human studies and clinical trials on the use of citicoline strongly support a role for citicoline as coadjuvant treatment for cognitive impairments in dementia, chronic degenerative diseases (including AD), stroke and cerebral ischemia and traumatic head injury [54,61,62,63]. Citicoline seems to be effective in improving cognitive abilities in normal healthy people as well [64].

**Selenium** (Se) is an important trace element involved in a number of important physiological functions [65,66,67]. It has been demonstrated that a decrease in the plasma concentrations of selenium is related to cognitive decline [68] and several reports show reduced levels of Se in the plasma of AD patients [69,70,71]. Hence, Se levels appear important for proper cognition and Se supplementation appears to hold potential for AD prevention and therapy. The relevance of Se to AD is not without a ground and relies on its strong antioxidant properties not in the last place stemming from the element being indispensable in enzymes such as glutathione peroxidases (GPx1-4, GPx6), thioredoxine reductases (TrxR1-3), selenoprotein P (SelP) and other selenoproteins, involved in reducing oxidative damage to cells and contributing to their oxidative stress defense [72]. Additionally, many studies reveal that Se can alleviate AD-associated pathology by interfering with both the formation of amyloid-β (Aβ) peptide plaques and neurofibrillary tangles, by affecting AD-disordered neurotransmitter metabolism and influencing AD-related signal transduction [72,73,74,75,76,77].

**Olive leaf** extract (OLE) has robust antioxidant and anti-inflammatory properties due to a remarkable content of bioactive phenols such as phenolic acids, phenolic alcohols, secoiridoid derivatives, flavanols, flavones, flavonols and others, amongst which oleuropein and hydroxytyrosol, in which OLE is particularly abundant, and with proven favorable effects regarding AD [78]. Indeed, plentiful research shows that OLE and the bioactive phenols in it are capable of preventing and reducing deteriorating changes related to AD by decreasing the total Aβ brain levels due to increased clearance and reduced production of Aβ, by diminishing neurofibrillary tangles formation, by inhibiting choline esterases, by decreasing oxidative stress and reducing neuroinflammation by inhibiting the NF-κB pathway and upregulating SIRT1, by enhancing autophagy and restoring loss of proteostasis, reflected in lower toxic protein aggregation [78,79,80,81].

**Green tea** with a multitude of bioactive compounds such as catechins, caffeine and theanine and particularly epigallocatechin gallate (EGCG), the main catechin in green tea, has also been targeted as a promising option for AD modulation. Catechins, and especially EGCG, have been proven to act as powerful antioxidant systems functioning in several different (additive) ways. First, they directly neutralize ROS, reactive nitrogen species and other free radicals with the antioxidant capacity of EGCG being even greater than that of vitamin E and C, thanks to a number of free OH groups available to sequester radicals. Second, they show indirect antioxidant effects by increasing the activity of SOD and catalase and by binding to antioxidant regulatory elements, inducing the expression of stress response genes relieving oxidative stress. In addition to its potent antioxidant effects, EGCG reveals an overabundance of other synergistic actions that shape its neuroprotective role, including reducing tau hyperphosphorylation and its aggregation; preventing the formation of Aβ and its subsequent accumulation by facilitating the nonamyloidogenic processing of the amyloid precursor protein (APP); chelating copper and iron thus preventing iron-mediated promotion of amyloid and tau pathology; modulating multiple intracellular signaling pathways related to neuroprotection and cell survival and associated with AD such as the protein kinase C (PKC), mitogen-activated protein kinases (MAPK) and phosphoinositide 3-kinase (PI3K/Akt) pathways [82]. 

**Vitamin D** is largely recognized for its effect on calcium and bone metabolism, but it has neuroprotective, antioxidant and anti-inflammatory properties as well. Some of the mechanisms by which calcitriol supports the normal brain function and exerts neuroprotective effects include upregulation of the synthesis of neurotrophins such as nerve growth factor (NGF), neurotrophin 3 (NT3) and glial cell line-derived neurotrophic factor (GDNF); modulation of neuronal Ca^2+^ homeostasis by downregulation of L-type voltage-sensitive Ca^2+^ channels and induction of the synthesis of Ca^2+^-binding proteins, such as parvalbumin and calbindin-D28K, protecting neurons from calcium excitotoxicity and neuronal death [83,84] and a reduction in glutamate neurotoxicity [85,86]. Furthermore, calcitriol has been reported to inhibit the synthesis of inducible nitric oxide synthase (iNOS), thus preventing excessive NO production and generation of toxic hydroxyl and nitrogen dioxide radicals; to increase GSH levels and to upregulate γ-glutamyl transpeptidase activity with a crucial role in maintaining GSH homeostasis [87]; to decrease the secretion of many inflammatory cytokines including IL-2, IL-6, IFN-γ and others and these are just a few of the calcitriols many antioxidant and anti-inflammatory properties. In relation to AD, numerous research using cellular and rodent models has shown that vitamin D relieves AD pathology by reducing Aβ accumulation through decreasing Aβ formation and increasing its degradation and by inhibiting tau phosphorylation accompanied by prolific cellular changes reducing neuronal loss, oxidative stress and neuroinflammation [88,89]. In humans, vitamin D levels have been found to positively correlate with memory and cognition and vit D deficiency has been demonstrated and postulated to be a risk factor for dementia and AD [90,91,92,93,94,95]. Several recent meta-analysis reports provide additional comprehensive evidence for low vitamin D levels presenting an increased risk for developing both dementia and AD and secure a place for the vitamin in AD preventive and adjuvant therapy especially in vitamin D deficient patients [96,97,98].

In view of the described in detail numerous versatile beneficial effects of the above substances (confirmed for LA in studies of ours as well [26,27]) underlined by complementary mechanisms of action, the aim of the present study was to study the effect of an experimental combination consisting of α-lipoic acid, citicoline, extract of leaves of green tea, extract of leaves of olive tree, vitamin D3, selenium, and an immune-supporting complex in an experimental model of Alzheimer’s-type dementia in rats as a possible part of an AD supporting therapy.

## 2. Materials and Methods

### 2.1. Experimental Combination

The original experimental combination was developed by our team and was patented (patent document No. 4391 U1 from 27 October 2022). It contains seven natural bioactive components, namely α-lipoic acid (Productos Quimicos Gonmisol S.A., Barcelona, Spain), citicoline (as Cognizin^®^, a trade mark by Kyowa Hakko Bio Co., Ltd, Tokyo, Japan), extract of leaves from green tea *Camellia sinensis*, L. (Plantex, Saint-Michel-sur-Orge France), extract of leaves from olive tree *Olea europea,* L. (Plantex, Saint-Michel-sur-Orge France), vitamin D_3_ (ProTec Nutra Ltd., Borehamwood, UK), selenium (as L-selenomethionin—Lallemand, Montréal, Canada/Europe) and an immune-supporting complex (Bulgarian patent (Bul Bio-National Center for Infectious and Parasitic Disesases, Sofia, Bulgaria). The overall technology for preparing the combination is ensured by NATSTIM (www.natstim.eu (accessed on 20 October 2023).

### 2.2. Laboratory Animals

The experiments were conducted on male adult Wistar rats (160–180 g), bred in the Experimental Breeding Facility, Slivnitsa, Sofia, Bulgaria. The animals were further transported and habituated to the conditions of the local vivarium at Institute of Neurobiology, Bulgarian Academy of Sciences, in plastic cages (n = 5) and under standard laboratory conditions—12 h light/dark cycle, unrestricted access to water and food, optimal temperature, humidity and ventilation of the rooms.

All experimental procedures were conducted in conformity with the institutional guidelines (Bioethics Committee at the Institute of Neurobiology, Bulgarian Academy of Sciences, Bulgaria) in compliance with the national and international laws and policies (the new Directive 2010/63/EU 22.09.2010) of the European Parliament and the Council of the European Union replacing the older Council Directive (86/609/EEC) on the protection of animals used for scientific purposes; the National Institute of Health (NIH) Guide for the Care and Use of Laboratory animals, NIH Publication No. 85–23, 1985 and Bulgarian laws (Ordinance No. 20 of 1 November 2012 on the minimum requirements for the protection and welfare of experimental animals and the facilities for their use, breeding and/or supply, issued by the Ministry of Food and Agriculture, Official Gazette No. 87 of 9 November 2012 and in force from 1 January 2013.

### 2.3. Experimental Groups

Rats were divided into the following experimental groups with 10 animals each:

(i) Control group (control)—receiving tap water daily orally (0.5 mL/100 g) for 51 days and at the end of the treatment, together with saline (0.5 mL/100 g, i.p.) for 11 days;
(ii) Scopolamine group (Sco)—receiving tap water daily orally (0.5 mL/100 g) for 51 days and at the end of the treatment, together with 2 mg/kg scopolamine (0.5 mL/100 g, i.p.) for 11 days;
(iii) Lipoic acid group (Sco+LA)—receiving LA (daily orally (100 mg/kg; 0.4 mL/100 g) for 51 days and at the end of the treatment together with injected scopolamine 2.0 mg/kg for 11 days;
(iv) Citicoline group (Sco+citicoline)—receiving citicoline daily orally (100 mg/kg 0.5 mL/100 g) (51 days) and at the end of the treatment together with injected scopolamine 2.0 mg/kg for 11 days;
(v) Lipoic acid and citicoline group (Sco+LA+citicoline)—receiving for 51 days the combination LA + citicoline daily orally (respectively 100 mg/kg, 0.4 mL/100 g LA and 100 mg/kg; 0.5 mL/100 g citicoline) and at the end of the treatment together with injected scopolamine 2.0 mg/kg for 11 days;
(vi) Experimental combination group (Sco+E)—receiving for 51 days the combination of α-lipoic acid, citicoline, extract of leaves of green tea, extract of leaves of olive tree, vitamin D3, selenium, and an immune-supporting complex (in the results and figures indicated as experimental combination, E) orally (0.5 mL/100 g) and at the end of the treatment together with injected scopolamine 2.0 mg/kg for 11 days.

### 2.4. Experimental Design

All behavioral, biochemical and histological changes induced by the experimental combination, lipoic acid, citicoline and the combination of lipoic acid and citicoline were evaluated using a model of Alzheimer’s type dementia in rats. Experimental dementia of Alzheimer’s type was chemically induced by repeated administrations of scopolamine hydrobromide (11 days, 2.0 mg/kg intraperitoneally) in laboratory male Wistar rats based on previous studies of ours [28].

The experimental combination as well as LA, citicoline and LA and citicoline together were applied daily orally in the corresponding doses for 40 days before and 11 days simultaneously with Sco (i.e., 51 days).

**Scheme 1:**
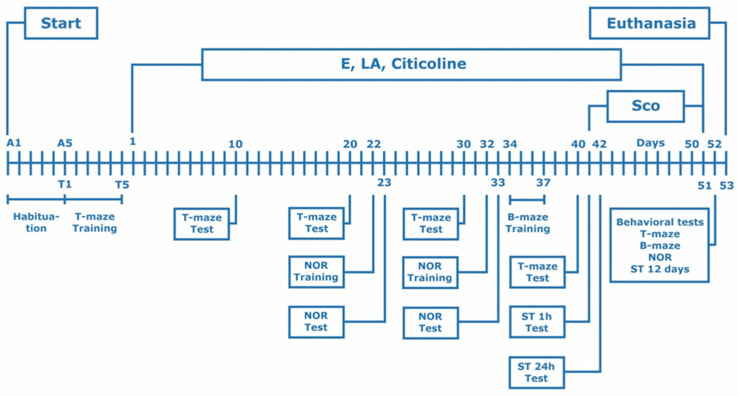
Schematic timeline of the experimental paradigm.

In the course of the experiment, several behavioral tests such as step-through inhibitory avoidance, T-maze, Barnes maze, novel object recognition were conducted (Scheme 1).

On the day after behavioral tests were performed (12th day from the start of scopolamine treatment), rats were euthanized under CO_2_ inhalation.

C-reactive protein and parameters of toxicity: aspartate aminotransferase (ASAT), alanine aminotransferase (ALAT), creatinine, albumin, globulin, total protein, were determined in blood serum/plasma of the rats. 

The internal organs of animals—liver, kidneys, small intestines and stomach—were taken for histological examination and for evaluation of possible toxic effects of the substances.

Brains were quickly removed and two brain structures related to memory—hippocampus and prefrontal cortex were carefully isolated on ice and used for biochemical studies: acetylcholinesterase (AChE) activity, oxidative stress and antioxidant defense system (LPO; levels ot tGSH, SOD catalase and GPx activities) brain-derived neurotrophic factor (BDNF) and phosphorylated cAMP response element-binding protein (pCREB) were assessed

### 2.5. Behavioral Experimental Methods

Several behavioral tests were used to evaluate changes in the memory and learning abilities of the experimental animals after application of scopolamine and to assess the restoring capacity of the experimental combination and its components.

#### 2.5.1. Step-Through Passive Avoidance

The test is one of the most commonly used to assess changes in learning and memory in rodents and especially suitable to study both the animals’ short and long-term memory as recall can be monitored (tested) easily in a one-trial session, at different times after acquisition [99].

The apparatus consists of two chambers, a dark and an illuminated one, separated by a guillotine door. The floor of the dark chamber is lined with a still grid for delivering electric shocks whenever the animal has stepped with all four paws in the chamber. The experiment was conducted in two phases (sessions), a training and a test session as described previously [23]. In the training session, preceding the start of scopolamine injections, rats were placed in the illuminated chamber and, after a habituation period of 1 min, by opening the guillotine door, allowed to enter the dark chamber where they received an unescapable electric foot shock (0.7 mA, 3 s, once). The time the animals needed to enter the dark chamber was recorded as initial latency (IL). In the test session, performed at the 1st and 24th hour and at the 12th day after scopolamine injections, rats were placed again in the illuminated chamber and again allowed to enter the dark compartment but without receiving a foot shock. The time the animals needed to enter the dark chamber was recorded as step-through latency (STL) (cut-off time 180 s) and used to evaluate memory and learning impairments in the different experimental groups.

#### 2.5.2. T-Maze

The test is particularly appropriate for studying specific aspects of spatial working memory and is less stressful compared to similar tests like the Moris water maze [100], working memory defined as short-time encoding of spatial information to adjust subsequent behavior. Dopaminergic signaling in the prefrontal cortex (PFC), the hippocampus and the striatum has been identified to be involved in the modulation of working memory [101]. The T-maze consists of a starting arm (50 × 10) and two other arms (40 × 20) arranged in a T-shape. It is based on the spontaneous or food-stimulated alternation of the T-shaped arms of the maze as animals enter them. 

In the second variant, used by us, the innate tendency of the animals to spontaneously alternate shoulders was stimulated by sequentially placing food in the two arms of the maze. This study was conducted in two phases (stages)—“training”—before the start of application of any of the tested substances (pretreatment) and a “test phase” on the 10th, 20th, 30th, 40th day of the pretreatment and on the 12th day after the application of scopolamine following a described protocol [23] using a modified protocol of [102]. Briefly, after a few days when the animals were adjusting to the experimenter’s touches (handling), in the next 5 days (“training”), they were habituated to the T-maze and prepared for the subsequent test phase. On the first day, the animals were allowed to freely explore the maze for about 15 min, with small pieces of food scattered across the maze at short distances to prompt the rodent to associate the maze with food and to motivate it to move forward after being placed at the base of the starting arm. On the second day, the food was only located at the end of the two T-shaped arms to motivate the animal to choose and enter one of those arms. In the most important part of the training, for three consecutive days, each day, the following experiment was conducted (consisting of 10 trials for each animal): the animal was placed at the base of the maze, in a compartment with a sliding (guillotine) door, and after 10 s released to spontaneously enter one of the T-shaped arms of the maze. In the first “forced trial”, the animal could only enter one arm (the other was closed with a guillotine door) and this was the arm where the food was located. In the following nine trials, both arms were accessible to the animal, but food was to be found only in the arm opposite to the one in which the food was in the previous trial. Entries into a T-arm were recorded as successful if the arm was with food and as unsuccessful if in the opposite arm. If the animal made an unsuccessful entry, it was allowed to continue exploring the maze until finding the food in the opposite arm and eating it. Calculated and compared between groups was the number of successful entries (correct choices) divided by all trials (10) and multiplied by 100 (T-maze performance in %). The ‘test phase’ on the corresponding day of the experiment comprised 10 trials and was conducted as described above.

#### 2.5.3. Barnes Maze (B-Maze)

The test is frequently used to evaluate changes in spatial learning and memory in rodents [103]. It employs an open, circular platform with a diameter of 122 cm, located 80 cm above the ground, holding 20 holes situated regularly at the platform periphery with a dark box for the animal’s escape under one the holes. A repulsive (aversive) stimulus of strong light (3000 lx) and loud noise (76 dB) was applied to encourage the animal to escape from the open space of the platform, (where placed during the experiment), and hide in the escape box. The evaluation of spatial memory was carried out by comparing the times it took the animals to find and enter the hole with the (underneath) escape box (total latency). The experiment was run, in two sessions, a “training” and a “test” session, following, with slight modifications, a previously described protocol [28]. The “training” was performed immediately before the start of the scopolamine injections or, for a group of animals, on the fourth day of scopolamine injections, and the “test session”—on the 12th day after the administration of scopolamine. The training took place over 4 days and during the first day the animals were placed in the center of the platform and allowed to explore it freely for 5 min. During the next 3 days, each day, 4 trials were conducted for each animal, 15 min apart, with the animal being placed in the center of the maze in a transparent starting chamber, from which it was released after 30 s to move freely in the maze. The time to find and enter the hole with the escape box underneath was recorded. On the first of the three days, training was conducted without the aversive stimulus, and on the other two days with the strong light and loud noise being on. The test session (on the 12th day after scopolamine administration) was a repetition of the last day of training: 4 trial sessions of 15 min each for each animal, recording the time to find the escape box and the number of unsuccessful trials until it was reached. The maze and escape box, the location of which was important not to be changed, were regularly cleaned with 70% ethanol to prevent olfactory cues.

#### 2.5.4. The Novel Object Recognition (NOR)

This test is used to assess changes in non-spatial memory and is a measure of visual (object) recognition memory [104]. It is centered on the recognition of familiar and novel objects using the innate curiosity of rodents and their predisposition to explore the new and unfamiliar and on the fact that if the rat encounters a familiar and a new object it will spend less time at the familiar one.

The experimental set up is a dark plexiglass chamber (60 × 60 × 40 cm) in which the rat can move without any constraints. This study was conducted on two consecutive days. On the first day, the animal was gently placed in the chamber and, for 5 min allowed to freely explore it without any objects in it. On the same day, a second 5 min phase followed when the animal was again introduced in the chamber but with two identical objects (A+A) in it. On the following day was the actual testing, when the animal was placed in the same environment with two objects, one of which, however, was the old (familiar) one and the other was similar, but different (A+B). The time spent exploring the old and new object was recorded over a period of 5 min. A discrimination index as a measure of recognition memory (RI) was calculated using the formula:

RI = (N/(N + F)) × 100,

where N is the time exploring the new object B and F—the time exploring the familiar object A.

Recognition index values range from 0 to 100%, with RI > 50% indicating that animals remember and discriminated objects, and RI < 50 are indicative of recognition memory impairments.

### 2.6. Biochemical Methods

#### 2.6.1. Tissue Preparation

On the day after the behavioral tests were conducted, the animals were decapitated, brains were quickly removed on ice and cortex and hippocampus separated. Both structures priorly frozen at −25° were cut into smaller tissue fragments and weighted. Fragments were mixed with PBS (0.01 mol/L, pH 7.4) and homogenized (tissue weight (g): PBS (mL), volume 1:9) with a Potter-Elvehjem glass homogenizer with a Teflon pestle. Homogenized tissue was centrifuged for 5 min at 5000× *g* and the supernatant was used for determining acetylcholinesterase (AChE) activity, the levels of lipid peroxidation (LPO) and the content of total glutathione (tGSH), as well as to measure BDNF and pCREB. A portion of the same diluted homogenate was centrifuged for 20 min at 12,000× *g* and the resulting postmitochondrial supernatant was used for the determination of the antioxidant enzyme activities: superoxide dismutase (SOD), catalase (CAT) and glutathione peroxidase (GPx). All procedures were kept under temperature control, between 0° and +4 °C.

#### 2.6.2. AChE Activity

Assessed in the brain’s structures based on the Ellman’s method [105]. The Ellman’s method is a spectrophotometry-based assay in which the increase in the yellow color of thiocholine produced by the catalytic action of acetylcholinesterase when 5,5’-dithio-bis (2-nitrobenzoic acid (DTNB) is added, is followed. A volume of 100 µL of the supernatant was incubated with the Ellman reagent: 2.9 mL of 0.1 M phosphate buffer (pH 8), 100 µL of 0.1 M DTNB and 20 µL of 0.075 M freshly prepared acetylthiocholine iodide. 500 µL of the reaction mixture was injected into the Semi-auto Chemistry Analyzer (Shenzhen Mindray Bio-medical Electronics CO., LTD, Shenzen, China) and kinetics of the reaction were monitored for 3 min at 405 nm.

All biomarkers for oxidative stress were measured spectrophotometrically using kits, purchased from Sigma-Aldrich Co. LLC, St. Louis, MO, USA.

#### 2.6.3. Lipid Peroxidation

Lipid peroxidation was determined by Lipid Peroxidation (MDA) Assay Kit MAK085 and was expressed as nmoles malondialdehyde (MDA) mg/protein, using a molar extinction coefficient 1.56 × 10^6^ M^−1^ cm^−1^.

#### 2.6.4. Total Glutathione

Total glutathione concentration was measured using Glutathione Assay Kit CS0260. The reaction gave a yellow color compound with an absorption peak at 412 nm. The amount of glutathione was calculated from the reference standard and expressed as ng/mg protein.

#### 2.6.5. Superoxide Dismutase

Superoxide dismutase activity was measured by SOD Assay Kit-WST 19160. The water-soluble tetrazolium (WST-1) was reduced by the superoxide radicals, generated in the xanthine-xanthine oxidase system, to formazan. The inhibition of the WST-1 reduction was considered a measure of SOD activity as one unit was defined as the amount of enzyme needed to inhibit the WST reduction by 50%.

#### 2.6.6. Catalase

Catalase activity was determined by using Catalase Assay Kit CAT100. The absorption decrease at 240 nm corresponded to the decomposition of H_2_O_2_ and was a measure of the catalase activity. Enzyme activity was expressed as _Δ_A240/min/mg protein. 

#### 2.6.7. Glutathione Peroxidase 

Glutathione peroxidase activity was measured using Glutathione Peroxidase Cellular Activity Assay CGP1. The enzyme activity was expressed as units/mg protein using a molar extinction coefficient 6.22 mM^−1^ cm^−1^ for NADPH.

#### 2.6.8. Protein Concentrations

Protein concentrations were measured according to Lowry et al. [106] by using a standard curve for bovine serum albumin.

#### 2.6.9. BDNF

BDNF was measured using a commercially available sandwich ELISA kit (MyBioSource Inc., San Diego, CA, USA, Catalog No MBS457896). Briefly, 100 µL of BDNF standards (0.156–10 ng/mL) and samples was added to the BDNF pre-coated 96-well plate. The plate was covered and incubated for 90 min at 37° and, further, the liquid was removed from each well. Next, 100 µL of Detection Solution A (Catalog No MBS457896) was added to each well and the plate incubated for 45 min at 37°, followed by a wash for 1–2 min 3 times with a wash buffer. In the next step, 100 µL of Detection Solution B (Catalog No MBS457896) was added to each well and the plate incubated for 45 min at 37°. Further the plate was washed for 5 times with a wash buffer, followed by incubation with TMB Substrate Solution for 15–25 min. The addition of 3,3′,5,5′-tetramethylbenzidine (TMB) gave a start to the color reaction, stopped 10 min later with a Stop Solution, followed by the immediate recording of the absorbance at 450 nm (MR-96A microplate reader, Shenzhen, China). Samples and standards were run and BDNF concentrations calculated using a standard curve.

#### 2.6.10. pCREB

pCREB was measured using a commercially available sandwich ELISA kit (MyBioSource Inc., USA, Catalog No MBS1600625). Briefly, 50 µL of pCREB standards (0–12 ng/mL) and samples was added to the pCREB pre-coated 96-well plate. The plate was covered and incubated for 60 min at 37° and, further, the liquid was removed from each well. Next, 50 µL of Detection Solution A and 50 µL of Detection Solution B was added to each well and the plate incubated for 10 min at 37°. In the next step, 50 µL of Stop Solution was added to each well followed by the immediate recording of the absorbance at 450 nm (MR-96A microplate reader). Samples and standards were run and pCREB concentrations calculated using a standard curve.

#### 2.6.11. ALAT and ASAT Activities

Blood was collected in serum separator tubes, centrifuged at 3000 rpm for 10 min to obtain serum, which was stored at −20° in aliquots. The activity of the two enzymes was determined using Giesse Diagnostics kit, Italy (Catalog No 419407 for ALAT and No 419107 for ASAT). Liquid reagents and serums were left at room temperature (15–25°) before use. 1000 µL of Reagent A and Reagent B (volume 4:1) were pipetted into test tubes. Next, 100 µL serum was added and the mixture gently shaken and left at 37° for 5 min. 500 µL of the reaction mixture was injected into the Semi-auto Chemistry Analyzer and kinetics were monitored for 3 min at 340 nm, using water as a reference.

#### 2.6.12. Creatinine

Blood was collected in serum separator tubes, centrifuged at 3000 rpm for 10 min to obtain serum, which was stored at −20° in aliquots. The activity of creatinine was determined using Giesse Diagnostics kit, Italy (Catalog No 006007). Liquid reagents and serums were left at room temperature (15–25°) before use. 1000 µL of Reagent A and Reagent B (volume 1:1) was pipetted into test tubes. Next, 100 µL serum was added and the mixture was gently shaken and left at 37° for 30 s. A volume of 500 µL of the reaction mixture was injected into the Semi-auto Chemistry Analyzer and kinetics were monitored for 3 min at 340 nm, using water as a reference.

#### 2.6.13. Total Protein

Total protein was determined by using Reagent ready for use (Giesse Diagnostics kit, Italy, catalog No E020). Liquid reagents and serums were left at room temperature (15–25°) before use. 1000 µL of Reagent was mixed with 12.5 µL serum, gently shaken and left at 37° for 10 min. 500 µL of the reaction mixture was injected into the Semi-auto Chemistry Analyzer and read at 546 nm against a reagent blank.

#### 2.6.14. Albumin

Albumin was determined by using Reagent ready for use (BioSino Bio-Technology and Science Inc., Beijing, China, catalog No E019). Liquid reagents and serums were left at room temperature (15–25°) before use. 1500 µL of Reagent was pipetted into test tubes. Next, 5 µL serum was added and the mixture gently shaken and left at 37° for 5 min. 500 µL of the reaction mixture was injected into the Semi-auto Chemistry Analyzer and read at 630 nm against a reagent blank. Globulins were determined as globulins = total protein–albumin.

#### 2.6.15. C-Reactive Protein (CRP)

We used a commercially available sandwich ELISA kit (MyBioSource Inc., USA, catalog No MBS457896a). Blood was collected in serum separator tubes, centrifuged at 3000 rpm for 10 min to obtain serum, which was further used at a 500-fold dilution. Briefly, 100 µL of CRP standards (0.78–50 ng/mL) and samples were applied to the CRP pre-coated 96-well plate. The plate was covered and incubated for 60 min at 37° and, further, the liquid was removed from each well. Next, 100 µL of Detection Solution A was added to each well and the plate incubated for 60 min at 37°, followed by a wash for 1–2 min 3 times with a wash buffer. In the next step, 100 µL of Detection Solution B was added to each well and the plate incubated for 30 min at 37°. Further the plate was washed for 5 times with a wash buffer, followed by incubation with Substrate Solution for 10–20 min. The reaction was stopped with a Stop Solution, followed by the immediate recording of the absorbance at 450 nm (MR-96A microplate reader).

### 2.7. Histological Studies

After dissection and macroscopic examination of the thoracic and abdominal cavity of the rats, stomach, liver, both kidneys and small intestine were separated, and the materials were fixed in 10% neutral buffered formalin for 24 to 48 h. After samples were taken for histological analysis, they were placed in biopsy cassettes for further processing (DIAPATH model: DONATELLO FAST). For the purpose of this study, the materials were stained by routine histological staining with hematoxylin and eosin.

### 2.8. Statistical Analysis

The required sample size was calculated a priori using G*Power software, version 3.1.9.6 based on the following assumptions: an effect size (Cohen’s f) of 0.50, a two-tailed alpha error probability of 0.05, and a power (1—beta error probability) of 0.80, distributed across six groups. The calculated sample size was 10 animals per group (a total of 60). The calculated sample size was consulted and subsequently approved by the Institute’s Ethics Committee and adhered to the 3R principle for replacement, reduction and refinement in animal research.

The normality of the distribution of experimental variables was assessed using the Shapiro–Wilk and Kolmogorov–Smirnov tests. They did not show evidence of non-normality (*p* > 0.05). Guided by these results, and after a visual inspection of the histograms for variables and the corresponding QQ plots, it was decided to use a parametric test for further analysis. Results are presented as the mean values ± standard error of the mean (SEM). Statistical analysis was conducted using a one-way analysis of variance (ANOVA) with the fixed factor, comprising six levels: Controls, Sco, Sco+ALA, Sco+citicoline, Sco+ALA+citicoline, and Sco+E. When significant differences were identified by ANOVA, Dunnett’s post hoc test was employed for comparing the means of several treatment groups with a control group, effectively controlling the significance level for multiple comparisons. Dunnett’s test was applied once to compare experimental results against healthy controls and a second time against scopolamine-treated (demented) animals. To further understand the effect sizes, Cohen’s d was calculated for each comparison. It ranged within the interval 0.10–0.35.

## 3. Results

### 3.1. Behavioral Assessment of the Effects of the Experimental Combination and Its Components on Learning and Memory

The effect of the experimental combination and its components in step-through inhibitory avoidance, T-maze, Barnes maze and novel object recognition (NOR) tasks was examined.

**Figure 1:**
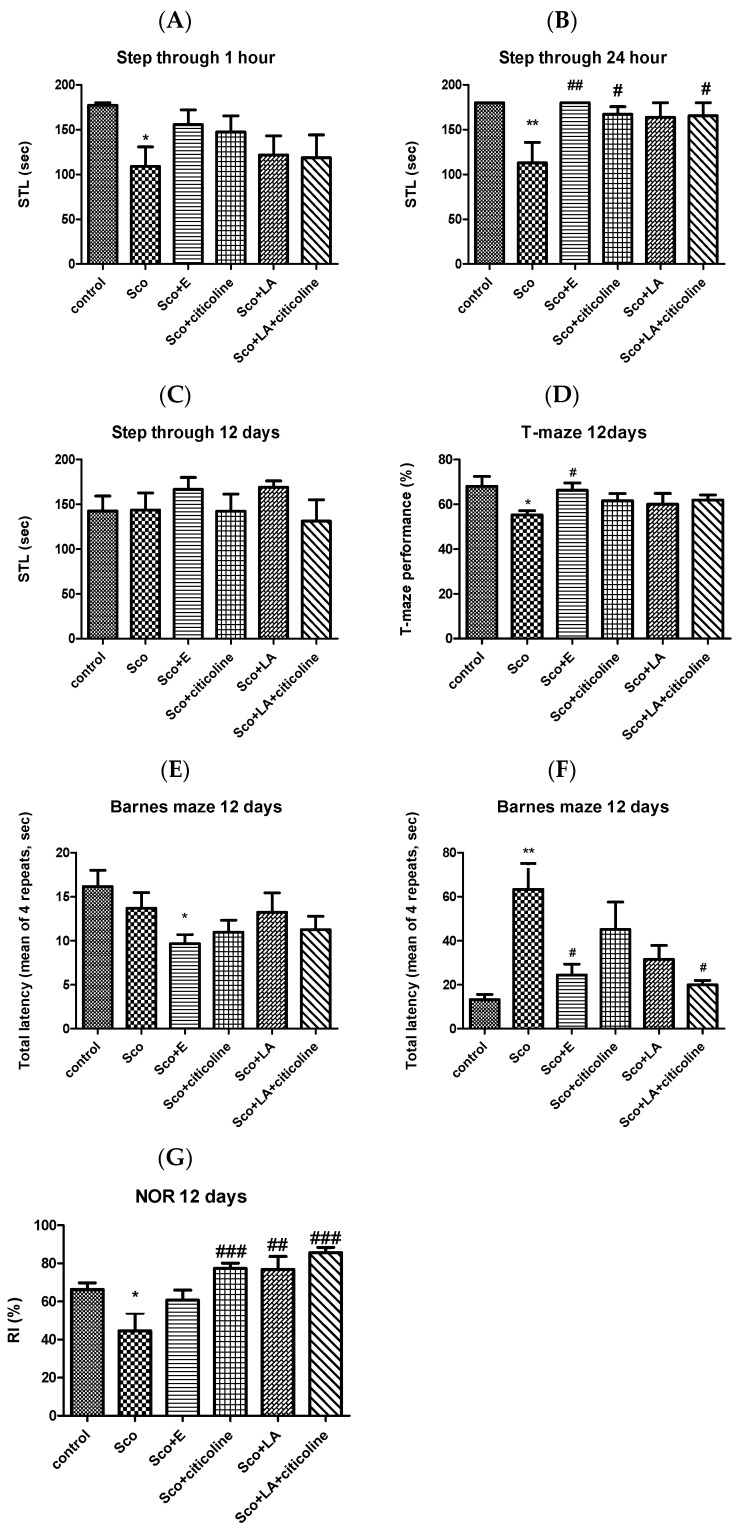
Effects of the experimental combination and its components, citicoline, alpha-lipoic acid (LA) and alpha-lipoic acid and citicoline (LA+citicoline) on learning and memory in a scopolamine model of dementia in rats. Step-through latency is assessed at 1 h, 24 h, and on the 12th day ((**A**–**C**), respectively) and T-maze performance is evaluated on the 12th day after the beginning of scopolamine treatment (**D**) for the control, Sco, Sco+E, Sco+citicoline; Sco+LA; Sco+LA+citicoline. Barnes maze total latency is assessed when Barnes maze training was performed before scopolamine treatment (**E**) and under scopolamine treatment (**F**). RI in the NOR study is evaluated on the 12th day after the beginning of scopolamine treatment (**G**). Data are expressed as the mean ± SEM; significant differences among experimental groups are denoted with “*” whenever groups are compared with the saline (control) group and denoted with “#” whenever groups are compared with the dementia (Sco) group. The number of “*” or “#” above bars is assigned as follows: # or * at *p* < 0.05; ## or ** at *p* < 0.01, ### *p* < 0.001.

Scopolamine treatment significantly decreases the step-through latency (STL) at 1 h and 24 h without having an effect on the 12th day after the beginning of scopolamine treatment (Figure 1A–C, respectively). At 1 h, all four groups (Sco+E; Sco+citicoline; Sco+LA; Sco+LA+citicoline) demonstrated no significant differences with the control group with, however, the group treated with the experimental combination showing STL values closest to those of the control group. At 24 h, the groups treated with citicoline, the combination of LA and citicoline and the experimental combination showed a significant difference with the scopolamine-treated group increasing the STL values, but it was the group treated with the experimental combination that showed the highest difference with the scopolamine-treated group the experimental combination practically completely restoring the decreased by scopolamine STL values. On the 12th day, the effects of all 5 groups were very much comparable with no significant differences between any of the groups, although STL values for the group treated with the experimental combination (and with LA) were highest.

Scopolamine significantly decreases the performance of the rats in the T-maze (Figure 1D). Although all four groups (Sco+E; Sco+citicoline; Sco+LA; Sco+LA+citicoline) demonstrated no significant differences with the control group, it was the group treated with the experimental combination that showed a significant difference with the scopolamine-treated group and it was the experimental combination that practically completely restored the impaired by scopolamine T-maze performance.

Figure 1E, F present the effect on the Barnes maze performance of the experimental combination and its components when the Barnes maze training was carried out before scopolamine treatment (E) and when the training was carried out under scopolamine treatment (starting on the 4th day of scopolamine treatment) (F). In the first case, scopolamine did not change the total latency, neither did citicoline, LA or the two antioxidants together. However, the experimental combination significantly reduced the total latency compared to control showing its favorable effect on spatial memory. In the second case, scopolamine significantly increased the total latency that was restored to control levels by all four groups, but most efficiently by the experimental combination and LA+citicoline which groups showed a significant decrease in total latency values compared to the scopolamine group.

Figure 1G demonstrates that scopolamine treatment resulted in significant lowering of the RI values in the NOR test and significant impairments of recognition memory as an RI index lower than 50% indicates recognition memory decline. In all four groups, this decline was effectively counteracted as those groups showed no differences in their RI values compared to the control group. The best effect was shown by the combination LA and citicoline which most efficiently increased RI values showing (out of all other groups) the biggest difference with the scopolamine group. 

### 3.2. Biochemical Assessment of the Effects of the Experimental Combination and Its Components on Learning and Memory

The effect of the experimental combination and its components on AChE activity, on malondialdehyde (MDA) levels as a measure of lipid peroxidation (LPO), on total GSH levels, on SOD, CAT and GPx activities as well as BDNF and phosphorylated CREB (pCREB) levels was measured.

#### 3.2.1. Effect of the Experimental Combination and Its Components on Brain AChE Activity

**Figure 2:**
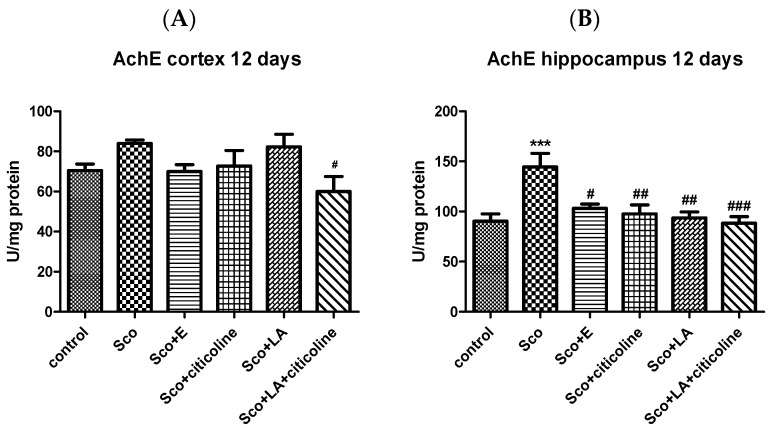
Effects of the experimental combination and its components, citicoline, alpha-lipoic acid (LA) and alpha-lipoic acid and citicoline (LA+citicoline) on AChE in the cortex (**A**) and hippocampus (**B**) of scopolamine-treated rats. Data are expressed as the mean ± SEM; significant differences among experimental groups are denoted with “*” whenever groups are compared with the saline (control) group and denoted with “#” whenever groups are compared with the dementia (Sco) group. The number of “*” or “#” above bars is assigned as follows: # at *p* < 0.05; ## at *p* < 0.01; ### or *** at *p* < 0.001.

The experimental combination and its components inhibited the scopolamine-increased acetylcholinesterase activity in rats with Alzheimer’s type dementia, both in the cortex and in the hippocampus (Figure 2). More pronounced were the effects in the hippocampus, where both the AChE-increasing effect of scopolamine and, in turn, the AChE-decreasing effects of the experimental combination and its components reached statistical significance. The decrease in the scopolamine-induced increase in AChE activity was comparable for the experimental combination and its components.

#### 3.2.2. Antioxidant Potential of the Experimental Combination and Its Components

LPO and tGSH levels as well as SOD, CAT and GPx activities were measured.

##### Effect of the Experimental Combination and Its Components on Brain LPO and tGSH Content 

**Figure 3:**
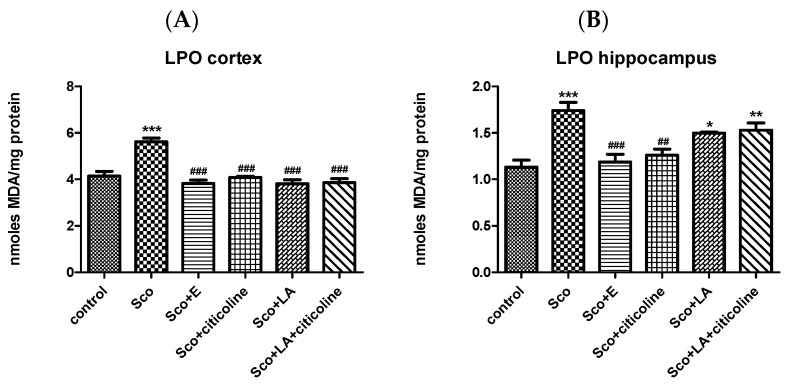

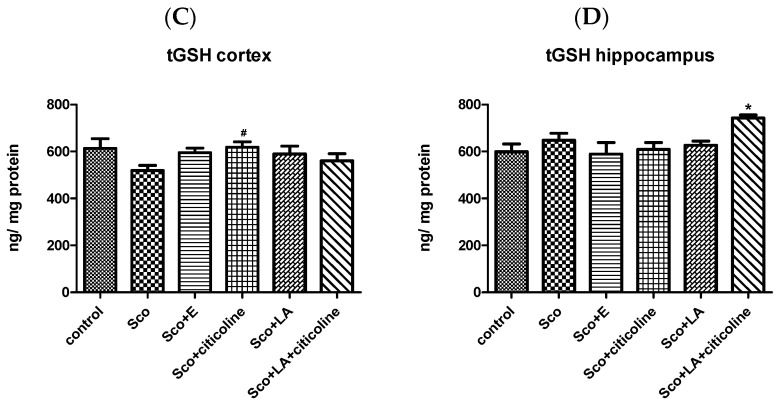
Effects of the experimental combination and its components, citicoline, alpha-lipoic acid (LA) and alpha-lipoic acid and citicoline (LA+citicoline) on the MDA levels (as a measure of lipid peroxidation, LPO) and tGSH in the cortex (**A**,**C**) and hippocampus (**B**,**D**) of scopolamine-treated rats. Data are expressed as mean ± SEM; significant differences among experimental groups are denoted with “*” whenever groups are compared with the saline (control) group and denoted with “#” whenever groups are compared with the dementia (Sco) group. The number of “*” or “#” above bars is assigned as follows: # or * at *p* < 0.05; ## or ** at *p* < 0.01; ### or *** at *p* < 0.001.

The level of MDA, thus LPO, was significantly increased by scopolamine in both cortex and hippocampus (Figure 3A,B). In the cortex, all four groups (Sco+E; Sco+citicoline; Sco+LA; Sco+LA+citicoline) showed no significant differences with the control group and a significant decrease compared to the scopolamine group. The effects of the experimental combination, citicoline and lipoic acid alone and the combination of citicoline+lipoic acid on scopolamine-increased MDA levels, were almost identical. In the hippocampus, in the groups Sco+E and Sco+citicoline, the MDA levels were restored to control levels and both groups showed no significant differences with the control group and a significant decrease compared to the scopolamine group. The strongest beneficial effect was held by the experimental combination showing the biggest difference with the scopolamine group.

A lower content (although not reaching statistical significance) of the tGSH in the cortex of the rats from the scopolamine group in comparison to the control group was observed (Figure 3C). In the other four groups (Sco+E; Sco+citicoline; Sco+LA; Sco+LA+citicoline), the level of the tGSH was comparable with that of the control group. The Sco+citicoline group stands out, showing a significant increase in tGSH levels compared with the scopolamine-treated group. In the hippocampus, the level of tGSH was comparable in the different groups with only the group treated with the combination of LA and citicoline showing significantly elevated tGSH levels compared to control animals (Figure 3D).

##### Effect of the Experimental Combination and Its Components on SOD, Catalase and GPx Activity

**Figure 4:**
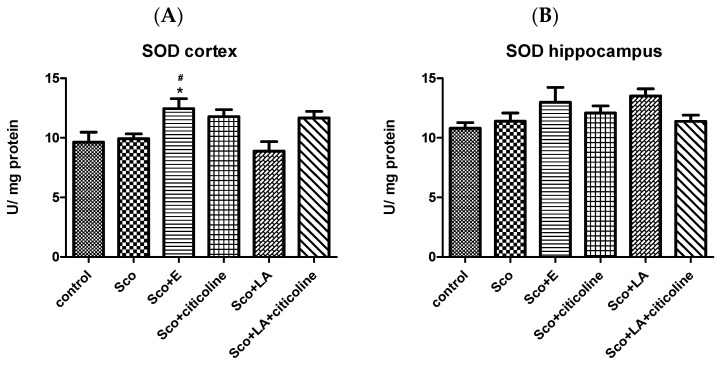

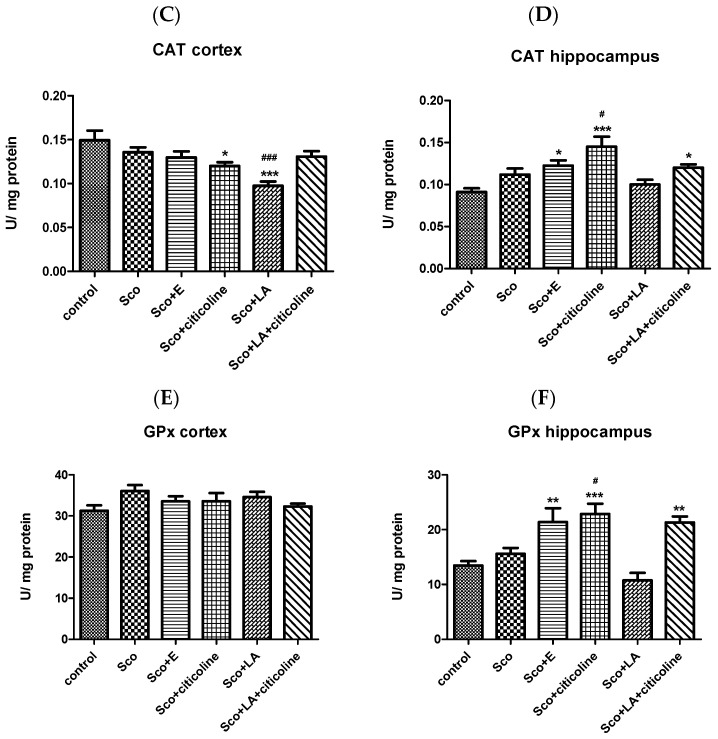
Effects of the experimental combination and its components, citicoline, alpha-lipoic acid (LA) and alpha-lipoic acid and citicoline (LA+citicoline) on SOD, catalase and GPx activity in the cortex (**A**,**C**,**E**) and on SOD, catalase and GPx activity in the hippocampus (**B**,**D**,**F**) of scopolamine-treated rats. Data are expressed as mean ± SEM; significant differences among experimental groups are denoted with “*” whenever groups are compared with the saline (control) group and denoted with “#” whenever groups are compared with the dementia (Sco) group. The number of “∗” or “#” above bars is assigned as follows: # or * at *p* < 0.05; ** at *p* < 0.01; ### or *** at *p* < 0.001.

SOD activity was not statistically significantly different between the scopolamine and control group in both cortex and hippocampus. In both cortex and hippocampus, however, SOD activity was higher in the group treated with the experimental combination reaching statistical significance in the hippocampus when compared to both the control and scopolamine group (Figure 4A,B).

Both in the cortex and hippocampus, no significant differences in the CAT activity of the scopolamine-treated and control groups were measured (Figure 4C,D). From the tested substances and their combinations, however, in the cortex, the treatment with citicoline and especially with LA led to a significant decrease in the CAT activity compared to the control group (also compared to the scopolamine group in the case of LA). In the hippocampus, the treatment with the experimental combination; the combination of LA and citicoline and, especially, with citicoline, lead to a significant increase in the CAT activity compared to the control group (also compared to the scopolamine group in the case of citicoline).

In the cortex, GPx activities remained unchanged in all groups (Figure 4E). In the hippocampus, however, a statistically significant increase (elevation) in the GPx activity was observed in the groups treated with the experimental combination, the combination of citicoline and LA and especially with citicoline (Figure 4F). In the latter case, the increase was significant not only compared to the control group, but compared to the scopolamine-treated group as well).

#### 3.2.3. Effect of the Experimental Combination and Its Components on the Brain BDNF/CREB Signaling Pathway

**Figure 5:**
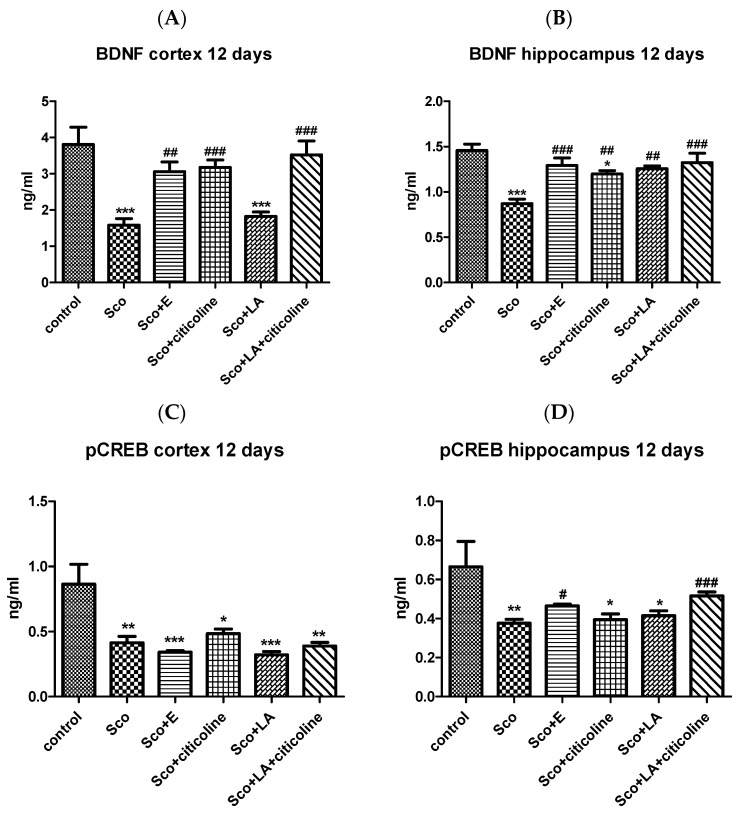
Effects of the experimental combination and its components, citicoline, alpha-lipoic acid (LA) and alpha-lipoic acid and citicoline (LA+citicoline) on BDNF levels in the cortex and hippocampus (**A**,**B**) and on pCREB levels in the cortex and hippocampus (**C**,**D**) of scopolamine-treated rats. Data are expressed as mean ± SEM; significant differences among experimental groups are denoted with “*” whenever groups are compared with the saline (control) group and denoted with “#” whenever groups are compared with the dementia (Sco) group. The number of “*” or “#” above bars is assigned as follows: # or * at *p* < 0.05; ## or ** at *p* < 0.01; ### or *** at *p* < 0.001.

Scopolamine significantly decreased BDNF levels both in the cortex and hippocampus (Figure 5A,B). In the cortex, the experimental combination, citicoline and the combination of citicoline and LA restored the decreased by scopolamine BDNF levels showing no difference with the control group and a significant difference with the scopolamine-treated group. LA failed to restore the decreased by scopolamine BDNF levels and the BDNF level in this group was comparable to the BDNF level in the scopolamine group. In the hippocampus, the experimental combination, LA and the combination of LA and citicoline returned BDNF levels to control values, with the best effect shown by the experimental combination and the combination of LA and citicoline demonstrating the biggest and most significant increase in BDNF levels compared to the scopolamine group.

Scopolamine significantly decreased pCREB levels both in the cortex and hippocampus (Figure 5C,D). In the cortex, none of the tested substances managed to restore pCREB values, with all four groups showing a significant decrease in pCREB levels compared to the control group. In the hippocampus, the experimental combination and the combination of lipoic acid and citicoline increased the decreased by scopolamine pCREB values and the groups treated with the experimental combination as well as with LA+citicoline showed no differences with the control group of animals. The groups treated with LA and citicoline alone showed pCREB levels similar to those of the scopolamine group and a significant decrease compared to the control group.

### 3.3. Histological Evaluation of Pathological Changes in Stomach, Small Intestine, Liver and Kidney of Male Wistar Rats Treated with the Experimental Combination and Its Components

Data for the innovative experimental combination were compared to those of the individual components. Results are shown in Table 1.

Table 1 presents the number of histopathological changes reflecting inflammatory processes, distributed by organs and studied groups (15 histological preparations per group were examined). Animals treated with scopolamine showed the highest number of lesions, while in the controls practically no inflammatory processes were observed.

Closest to healthy animals is the group treated with the experimental combination (only one inflammatory change in the liver). Rats treated daily with citicoline, lipoic acid, and the combination of citicoline and lipoic acid occupied an intermediate position, with seven, five, and eight pathological changes, respectively.

The evaluation of the pathological changes in the stomach, small intestine, liver and kidney of male Wistar rats treated long-term with the experimental combination recorded on the 12th day after the daily administration of scopolamine shows almost identical histological findings compared to the control group treated only with saline: the stomach lacks inflammatory changes, mucinous availability is reduced; in the small intestine, no significant differences were found with the controls—rate of changes (=0); no significant changes in the liver, except for a single initially forming intraparenchymal lymphoid aggregate—rate of changes (=1); in kidneys, no significant differences with the controls were found—rate of changes (=0).

Animals treated with the combination had the highest level of recovery from scopolamine, followed by those treated with lipoic acid alone, citicoline alone, and the combination of lipoic acid and scopolamine.

### 3.4. Biochemical Evaluation of Toxicity and Anti-Inflammatory Effects of the Experimental Combination and Its Components

The effect of the experimental combination and its components on ALAT and ASAT activities and on creatinine, albumin, globulin, total protein and CRP levels was measured.

No differences between any of the groups were observed for ASAT and ALAT activity and for creatinine, globulin and total protein levels. The only effect was observed for albumin where scopolamine significantly decreased the control levels with all the substances counteracting this change and all four groups showing no differences with the control group (Supplementary Figure S1).

**Figure 6:**
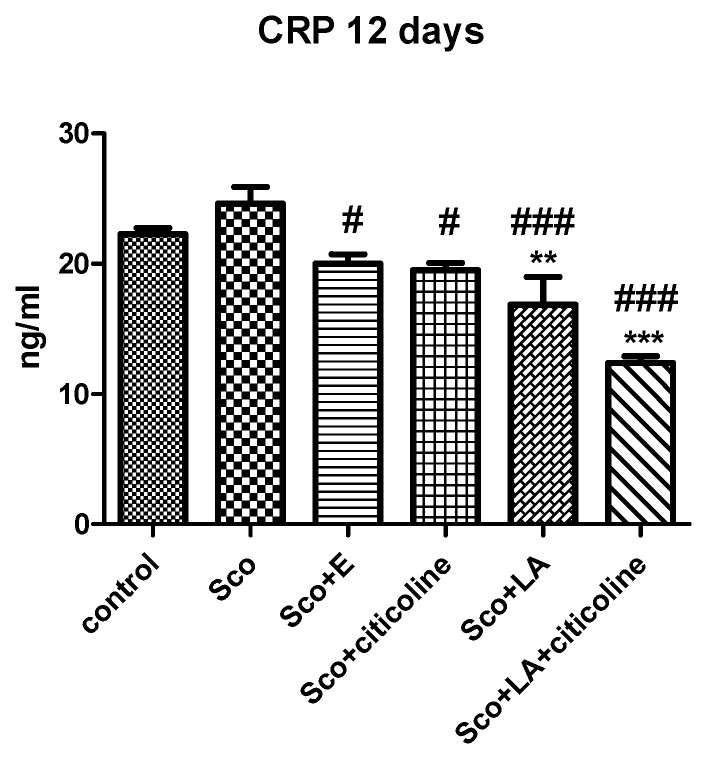
Effects of the experimental combination and its components, citicoline, alpha-lipoic acid (LA) and alpha-lipoic acid and citicoline (LA+citicoline) on CRP levels of scopolamine-treated rats. Data are expressed as mean ± SEM; significant differences among experimental groups are denoted with “*” whenever groups are compared with the saline (control) group and denoted with “#” whenever groups are compared with the dementia (Sco) group. The number of “*” or “#” above bars is assigned as follows: # at *p* < 0.05; ** at *p* < 0.01; ### or *** at *p* < 0.001.

Scopolamine showed no significant inflammatory effect with CRP levels in blood being only slightly increased in the scopolamine group of animals compared to the control group (Figure 6). It is interesting, however, that both the experimental combination and its components significantly reduced the level of CRP compared to the scopolamine animals and even to the control animals (for lipoic acid and the combination of lipoic acid and citicoline) indicative for a certain anti-inflammatory effect of all the substances. 

## 4. Discussion

In the present study, using behavioral and biochemical methods, we examined the effect on learning and memory and the neuroprotective effect of an experimental combination consisting of α-lipoic acid, citicoline, extract of leaves of green tea, extract of leaves of olive tree, vitamin D3, selenium, and an immune-supporting complex in a model of scopolamine-induced Alzheimer’s-type dementia in rats. We further evaluated the mechanism of the effect of the experimental combination as well as its toxicity and safety.

Scopolamine is a muscarinic cholinergic receptor (mACh Rs) antagonist, which causes memory and cognitive deficits (impairments) elicited by dysfunction of the brain cholinergic system, unfavorable changes in the monoaminergic neurotransmitter systems, increased oxidative stress and impaired antioxidant defense, mitochondrial damage, apoptosis, neuroinflammation and reduced BDNF-CREB signaling, pathological changes found in AD as well [4,5,6,7,8,9,107,108]. Furthermore, scopolamine contributes directly to AD pathology by stimulating Aβ generation and increasing Aβ levels as well as by increasing the amount of the phosphorylated tau protein [109,110,111,112]. The former effect is possibly carried out in part by hampering muscarinic transmission as treatment with selective muscarinic agonists has been shown to decrease cerebrospinal fluid levels of Aβ in AD patients [113,114]. Thus, we considered scopolamine a suitable tool to assess the value of the experimental combination both for memory and cognition and as a possible assisting therapy in AD.

Several behavioral methods such as step-through inhibitory avoidance, T-maze, Barnes maze and NOR (the novel object recognition task) were used to evaluate the changes in learning and memory induced by scopolamine and the capacity of the experimental combination and its components to counteract them. 

Step-through inhibitory avoidance is one of the most widely used behavioral methods to assess memory and cognition. The step-through inhibitory avoidance task is acquired through associative learning with an emotional and spatial memory component to it and is dependent on the integrated activity of several brain areas such as the entorhinal and posterior parietal cortex and hippocampus with dorsal hippocampus being of utmost importance in the acquisition, consolidation and retrieval of the rat’s passive avoidance response [115]. The activation of the cholinergic system through binding of ACh released by cholinergic fibers to muscarinic cholinergic receptors in the hippocampus plays a key role in inhibitory avoidance processes as administration of mAChRs antagonists, such as atropine, has been shown to worsen and of mAChRs agonists, such as oxotremorine or the muscarinic toxin MT2, to facilitate the acquisition of inhibitory avoidance tasks [116,117,118].

As a muscarinic cholinergic antagonist scopolamine administered intraperitoneally, intracerebrally or intrahippocampally has been plentifully shown to impair the performance of inhibitory avoidance tasks [119]. Our results showing scopolamine-induced decline in recall by significantly reducing the step-through latency at 1 h and 24 h is in agreement with data in the literature showing decrease in this parameter at 1 and 24 h both in mice and rats [18,120,121,122]. Interestingly, but not surprisingly, scopolamine did not impair the inhibitory avoidance task on the 12th day after daily scopolamine injections. This effect, which we observed in previous experiments as well, is corroborated by findings that different molecular mechanisms may underlie short-term and long-term memory formation [23,123]. In the present experiments, the experimental combination restored the STL values of scopolamine-treated rats to that of control rats at both 1 h and 24 h showing the best effect compared to citicoline and LA acid alone and the combination of LA and citicoline, especially at 24 h.

Various mazes are used to assess spatial learning and memory in rats. In our studies, we chose the T-maze that relies on positive reinforcement and the Barnes maze, that relies on repulsive agitation such as the combination of bright light and noise. Barnes maze is similar to the traditionally used Moris water maze, but avoids a strong aversive stimulus such as swimming as high levels of stress are known to influence the animals’ performance [124]. Spatial learning and memory are dependent on the hippocampus, on proper functioning of the cholinergic system and very much on the activation of the hippocampal M1 muscarinic cholinergic receptors. Indeed, tasks requiring spatial information processing and spatial memory formation are impaired in patients with hippocampal brain damage and animal studies have shown that hippocampal lesions produce deficits in performance of the water maze [125,126] and the radial arm maze [127] tasks. Furthermore, lesions of the cholinergic neurons in the medial septum providing cholinergic input to the hippocampus have been shown to impair both acquisition and maintenance of spatial memory in the T-maze [128], the water maze [129,130] and the radial arm maze [131,132] tasks. Finally, drugs acting selectively on M1 cholinergic receptors have been demonstrated to influence spatial-memory related tasks [133,134]. For example, the selective M1 muscarinic receptor antagonist pirenzepine significantly reduces the spontaneous alteration performance in an Y maze and McN-A-343, a selective M1 muscarinic receptor agonist, significantly improved the pirenzepine-induced impairment [133]. As an M1 muscarinic receptor antagonist, though not selective and thus with even broader effects on cognition, scopolamine has been shown to disrupt spatial learning and memory in the radial arm maze [135,136], in the Moris water maze [137] as well as the T-maze [14,28,138] and the Barnes maze [28,139]. In agreement with the literature, in our studies, scopolamine impaired both the T-maze and the Barnes maze tasks reflected in lower T-maze performance and higher total latency in the Barnes maze in scopolamine-treated rats. In the case of the Barnes maze task, the effect of scopolamine was significant only when training was carried out under scopolamine. This is in line with the finding that it is during acquisition of spatial learning, that ACh is released in the hippocampus and cortex (whereas during consolidation of spatial memory ACh levels remain low), underlining the importance for spatial memory formation of ACh receptor blockade during acquisition [140]. In all spatial memory tests, the experimental combination showed the best effects. In the T-maze test, although no differences in performance were observed between scopolamine-injected rats treated with the experimental combination, citicoline, LA or citicoline+LA and control rats, it was scopolamine-injected rats treated with the experimental combination that showed significantly higher performance compared to rats treated only with scopolamine and it was the experimental combination that restored the control values completely. In the Barnes maze, when scopolamine was applied post training, total latency was lowest (thus animals fastest to find the safety box) in the group treated with the experimental combination and the total latency values of this group were significantly lower even than the values of the control group. When training was performed under scopolamine and scopolamine significantly increased total latency it was again the experimental combination (but also citicoline) that most efficiently counteracted the effect of scopolamine, significantly decreasing the scopolamine-raised values and most effectively returning them to control values.

In addition to the effect on spatial memory, we evaluated the effect of the experimental combination on recognition memory using NOR. The test is used to assess changes in non-spatial memory and is a measure of visual (object) recognition memory. It is based entirely on the rats’ spontaneous behavior and can be considered a “pure” test for working memory without a reference component, dependent on the rodent’s natural exploratory instinct, in the absence of externally imposed rules or punishments [104]. Recognition memory involves processing of several types of information such as objects and locations, is mediated through several brain regions, especially the hippocampus and the perirhinal and medial prefrontal cortices and is distinct from spatial memory [141]. Similar to spatial memory, however, scopolamine impairs recognition memory too [142] as shown in our experiments as well. In addition to improving scopolamine-impaired spatial memory, the experimental combination is capable of restoring recognition memory as well by increasing the values of the discrimination index returning them to control values.

Scopolamine is well known to induce cholinergic system dysfunction. In addition to being a muscarinic receptor antagonist (and muscarinic cholinergic receptor activation has a central role in synaptic communication and plasticity, learning and memory formation and cognitive function [143,144,145]), it reduces acetylcholintransferase activity involved in the synthesis of ACh and increases AChE and butyrylcholinesterase activities, relevant to the degradation of ACh [146,147,148]. In agreement with data in the literature, our results also show increased AChE activity in the cortex and hippocampus of scopolamine-treated rats with the values in the hippocampus of scopolamine-treated animals being significantly higher than those of control rats. The experimental combination decreased AChE activity and returned it to control levels. AChE, being the enzyme that breaks down acetylcholine, is fundamental for correct cholinergic transmission and its excessive activity has been many times shown to be associated with cognitive and memory impairments in different experimental models [149,150]. However, excessive AChE activity is further detrimental by aggravating AD pathology by interacting with Aβ and promoting amyloid fibril formation and by forming AChE-Aβ complexes which are more harmful than the amyloid fibrils alone [151,152]. Thus, the alleviating effect of the experimental combination on the increase in AChE activity in scopolamine-treated rats explains its memory improving effect, but, even more important, prompts its use not only in conditions of memory and learning deficits, but as part of multifactorial AD treatment as well.

The beneficial effect of the experimental combination on memory and cognition in scopolamine-treated rats could be further explained with the observed decreased oxidative stress induced by decreases in lipid peroxidation and increases in CAT, GPx and SOD activities. 

Oxidative stress, defined as the imbalance between oxidant molecules and the organism antioxidant defense system and reflected in increased lipid peroxidation, generation of excessive free radicals and altered antioxidant enzyme activities, has been shown to be related to impairments in learning and memory and is considered part of the pathophysiological mechanism involved in the progression of cognitive dysfunction [153,154,155]. Oxidative damage along with accompanying enhanced lipid peroxidation and decreased activity of the major antioxidant enzymes such as SOD, CAT and GPx have been shown to be characteristic features of AD as well [156]. Furthermore, Esposito and colleagues have shown that in human amyloid precursor protein (hAPP) transgenic mice, inactivating one Sod2 allele (Sod2^+/−^) and reducing Sod2 activity exacerbates Alzheimer’s disease-like pathology and have suggested that increasing Sod2 activity might be of therapeutic benefit whereas Clausen and colleagues have explicitly demonstrated prevention of cognitive deficits and brain oxidative stress with superoxide dismutase/catalase mimetics in aged mice [157,158].

Our results show that the experimental combination is increasing the activity of SOD in the cortex and catalase and GPx activity in the hippocampus, which together with its demonstrated AChE-inhibiting ability is setting it as an attractive candidate in the prevention and treatment of cognitive (age) decline and AD. 

Although, in comparison with LA and citicoline alone and LA+citicoline, the experimental combination shows the most consistent effects in attenuating oxidative stress (for example, LA, compared to both control and even scopolamine, significantly decreases catalase activity in the cortex, rather than increasing it; citicoline also decreases catalase activity in the cortex), citicoline is also noticeable for its favorable effects, especially in the hippocampus. Indeed, in line with our results, data in the literature shows that citicoline is reducing oxidative damage in a variety of conditions (such as cerebral ischemia, lead-induced oxidative injury; arsenic-induced hepatotoxicity and others) including by decreasing lipid peroxidation and increasing the activity of catalase and GPx [52,159,160,161].

While it looks like the oxidative stress relief carried out by the experimental combination is mostly due to citicoline, its added value compared to the single antioxidant is certainly owed also to vit D, the green tea and olive tree leaf extract and certainly Se, as GPx enzymes are selenium-containing enzymes. Thus, it has been shown that in rats with spinal cord injury sodium selenite treatment greatly decreases the levels of the lipid peroxidation products malondialdehyde and 4-hydroxynonenal and increases the protein and mRNA expression of glutathione peroxidase 4 [162]. Regarding AD, it has been shown that selenomethionine (one of the main natural sources of Se and found in many food) is protective against Aβ and iron/hydrogen peroxide-mediated hippocampal neuronal death, an effect mediated by increased GPx protein and activity [163]. Vitamin D assists in antioxidation by upregulating the expression of glutathione and superoxide dismutase and downregulating the expression of NO and the inducible nitric oxide synthase (iNOS) [88, 164].

Green tea and olive tree leaf extracts have also been shown to favorably affect lipid peroxidation and SOD, CAT and GPx, exerting neuroprotective effects and reducing oxidative damage and possibly contributing to the overall effect of the experimental combination [165,166,167,168].

In recent years, cholesterol metabolism in the brain has been shown to be increasingly implicated in AD progression. A growing body of evidence indicates that oxidized cholesterol in the form of oxysterols plays a significant role in AD development enhancing oxidative stress and inflammation, as well as Aβ-formation and accumulation [169,170]. Oxysterols shown to be most closely related to AD are 24-hydroxycholesterol and 27-hydroxycholesterol, products of cholesterol oxidation by CYP46A1 and CYP27A1 and especially 7-ketocholesterol and 7β-hydroxycholesterol, resulting from cholesterol autooxidation. 7-ketocholesterol and 7β-hydroxycholesterol cause a specific form of cytotoxic activity defined as oxiapoptophagy, associated with organelle dysfunction and in particular with mitochondrial and peroxisomal changes involved in the induction of cell death and in the rupture of redox balance [171]. Epicatechin, epigallocatechin gallate (EGCG) and hydroxytyrosol, found in tea and olive tree leaf extracts have been demonstrated to counteract 7-ketocholesterol-induced cytotoxicity [171,172] reinforcing a further interest in the usage of the experimental combination in the treatment of AD.

Deciphering further the molecular mechanisms underlying the effect of the experimental combination, we examined the changes in BDNF in the cortex and hippocampus of scopolamine-treated rats and rats treated with the experimental combination and its main components. BDNF is an important neurotrophin involved in neurogenesis, neuronal differentiation and neuronal protection and crucial for learning and memory since it regulates synaptic function, synaptic plasticity and long-term potentiation in the hippocampus [107,173]. Accordingly, it has been shown that interference with the BDNF/TrkB signaling pathway either by genetic manipulations or pharmacological interventions causes memory and cognitive impairments and that chronic BDNF deficiency in BDNF knock out mice results in age-dependent learning deficits [174]. Evidence from both patients and animal models has suggested relation of BDNF to AD and its involvement in AD pathology as well. Thus, research has shown alterations in the BDNF levels in AD patients as well as low BDNF mRNA and protein levels in postmortem AD brain samples [107,108]. Regarding the role of BDNF in the disease amyloid or tau pathologies, although not completely elucidated, protective effect of BDNF against Aβ-induced neurotoxicity in vitro and in vivo in rats has been observed [175]. Scientific reports show that scopolamine decreases BDNF, which is in in agreement with our results, and demonstrates a significant reduction in BDNF levels both in the cortex and hippocampus of scopolamine-treated animals [18,137,176]. The experimental combination most consistently increases BDNF levels, restoring the neurothrophin control values both in the cortex and hippocampus. For comparison, although able in the hippocampus, in the cortex, LA fails to restore BDNF levels. Citicoline alone and in combination with LA is also restoring scopolamine-decreased BDNF values pointing to a significant role of citicoline in the BDNF-increasing effect of the experimental combination. This is not surprising as, recently, alone and in combination with Coenzyme Q and vit E, citicoline has been reported to exert complex protective effects in neuronal cells exposed to oxidative stress by reducing the gene expression proinflammatory factors such as of IL-6 and TNFα, and by inducing the gene expression of BDNF [177]. 

The binding of BDNF to its receptor tyrosine kinase TrkB leads to the dimerization and autophosphorylation of the receptor and subsequent activation of intracellular signaling cascades through which BDNF induces phosphorylation and activation of the cAMP response element-binding protein (CREB) [178]. Phosphorylation of the transcription factor CREB, the further recruitment of transcription cofactors such as CREB binding protein (CBP) and the following transcription of genes such as *Egr-1* are thought to play major roles in synaptic plasticity and long-term potentiation and thus in memory formation [179]. Similar to our studies in which scopolamine reduces the level of phosphorylated CREB, both in the cortex and hippocampus, other authors have also shown a scopolamine-induced reduction in pCREB expression [18,137,176]. Neither the experimental combination nor its components managed to restore control pCREB values in the cortex, whereas in the hippocampus the experimental combination and the combination of LA and citicoline significantly increased the reduced by scopolamine levels of pCREB so that no difference was observed between the control group and the group treated with the experimental combination and the combination of LA and citicoline.

Together with decreasing oxidative stress and reducing AChE activity, we believe it is, to a great extent, the upregulation of BDNF (and pCREB levels) in scopolamine-treated animals that underlies the memory improving effect of the experimental combination shown in all used behavioral tests (regarding both spatial memory revealed in the Barnes maze and the T-maze and the fear-motivated associative learning and memory revealed in the step-through passive avoidance test). Indeed, several lines of evidence point to a substantial part being played by BDNF and activation of its receptor TrkB in the hippocampus-dependent memory tasks such as the various mazes and the passive avoidance tests. First, the Morris water maze and passive avoidance tests are linked to increases in BDNF mRNA expression in the hippocampus [178,180]. Second, genetic manipulations in rodents causing BDNF deficiencies or defects in its receptor TrkB result in compromised hippocampus-dependent memory tasks performances. Thus, BDNF knockout mice show cognitive deficits in the T-maze [181] and BDNF mutant mice and mice overexpressing truncated trkB perform significantly worse in a Morris water maze task [182]. Functional blocking of BDNF, through anti-BDNF antibodies, for example, is again shown to be reflected in memory losses shown in the water maze and passive avoidance tests [183,184]. Additionally, CREB mutant mice and rats subjected to heat stress related to perturbations in the BDNF/ERK1/2/CREB axis show impaired hippocampus-dependent spatial memory [155,185].

In addition to examining the effects of the experimental combination on learning and different types of memory and unravelling the mechanisms of these effects through studies on AChE activity, oxidative stress and antioxidant defense system markers as well as BDNF/pCREB signaling, we assessed its toxicity and safety of use. Histological evaluation of the pathological changes in stomach, small intestine, liver and kidney of the experimental animals revealed no significant inflammatory processes in the rats treated with the experimental combination and showed histological findings almost identical with those in the control group treated only with saline. The lack of inflammation is also confirmed by the lack of changes in CRP in the group treated with the experimental combination compared to the control group of animals. Additional examination of blood biochemical parameters such as ALAT, ASAT, creatinine, albumin, globulin and total protein confirmed the lack of side effects on liver and kidney function and further excluded any inflammatory effects of the experimental combination. Actually, it is worth noting that the groups treated with the experimental combination and its components all showed significantly lower CRP values compared to the scopolamine-treated group, with the groups treated with LA and LA+citicoline showing even lower CRP values that those in the control group. 

**Figure 7:**
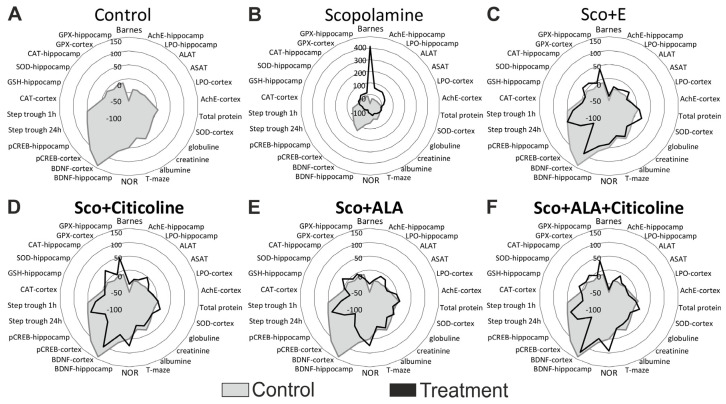
Radar plots showing the overall treatment profiles, represented as the percent change from baseline for each experimental parameter. A profile of healthy controls, depicted in gray, is superimposed on each treatment profile, depicted in black, to illustrate the extent of recovery or impairment (in the case of scopolamine treatment) for each parameter. (**A**) Profile of control treatment; (**B**) profile of scopolamine treatment; (**C**) profile of the treatment with scopolamine and the experimental combination (Sco+E); (**D**) profile of the treatment with scopolamine and citicoline (Sco+citicoline); (**E**) profile of the treatment with scopolamine and lipoic acid (Sco+ALA); (**F**) profile of the treatment with scopolamine and the combination of lipoic acid and citicoline (Sco+ALA+citicoline).

**In summary**, our study examined the effect of a unique combination of seven components—α-lipoic acid, citicoline, extract of leaves of green tea, extract of leaves of olive tree, vitamin D3, selenium and an immune-supporting complex, all of which with well-known and proven neuroprotective and memory and cognition preserving and enhancing properties. Compared to its components, the experimental combination showed the most complex beneficial effect in a scopolamine model of Alzheimer’s type dementia in rats. It efficiently restored various types of memory such as short-term and long-term memory (as judged by the step-through inhibitory avoidance test) as well as spatial (T-maze, Barnes-maze) and recognition memory (NOR) based on favorable effects as assessed by AChE activity, LPO, tGSH content, SOD, catalase, GPx activity, BDNF and pCREB, which on many occasions exceeded the effects of the separate components (Figure 7).

The multifaceted beneficial effects of the experimental combination underlined by the versatile and complementary mechanisms of actions of its components combined with lack of toxicity, antioxidant and anti-inflammatory effects make the experimental combination a suitable candidate for a multitarget cognition improvement and Alzheimer’s type dementia therapy.

## 5. Patents

The original experimental combination which we investigated in the present study was patented (document No. 4391 U1 from 27 October 2022).

## Figures and Tables

**Table 1:** Evaluation of pathological changes in stomach, small intestine, liver and kidney of male Wistar rats treated with the experimental combination.

Treatment	Number of Pathological Changes				
	Stomach	Small Intestine	Liver	Kidney	Total
Control	0	0	0	0	0
Sco	3	2	6	4	15
Sco+E	0	0	1	0	1
Sco+Citicoline	1	4	2	0	7
Sco+ALA	0	3	2	0	5
Sco+ALA+Citicolne	5	1	1	1	8

## Data Availability

The original data presented in the study are included in the article, further inquiries can be directed to the corresponding authors.

## Supplementary Materials

The following supporting information can be downloaded at: https://www.mdpi.com/article/10.3390/antiox12122050/s1, Figure S1: Effects of the experimental combination and its components, citicoline, alpha-lipoic acid (LA) and alpha-lipoic acid and citicoline (LA+citicoline) on ASAT and ALAT activity and creatinine, albumin, globulin and total protein levels of scopolamine-treated rats.
