# Supplementary material for: Memory Recovery Effect of a New Bioactive Innovative Combination in Rats with Experimental Dementia
Source: Antioxidants (Basel). 2023 Nov 28;12(12):2050. doi: 10.3390/antiox12122050 (PMC10740861; doi:10.3390/antiox12122050)
Supplement: Supplementary file 1 [file antioxidants-12-02050-s001.zip › antioxidants-2724041-supplementary.pdf]

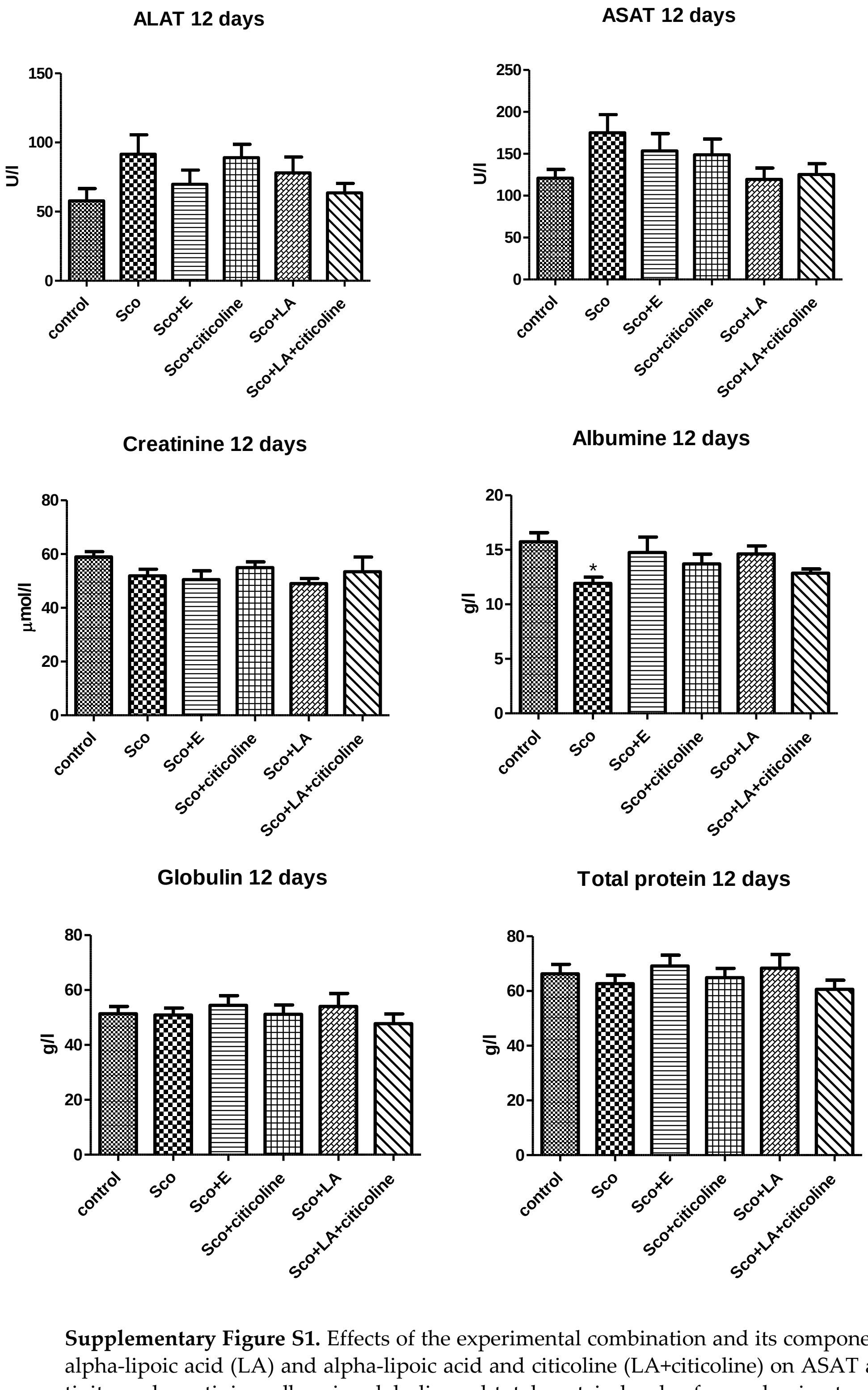

Supplementary Figure S1. Effects of the experimental combination and its components, citicoline, alpha-lipoic acid (LA) and alpha-lipoic acid and citicoline (LA+citicoline) on ASAT and ALAT activity and creatinine, albumin, globulin and total protein levels of scopolamine-treated rats. Data are expressed as mean±SEM; significant differences among experimental groups are denoted with "*" whenever groups are compared with the saline (control) group and denoted with "#" whenever groups are compared with the dementia (Sco) group. The number of "∗" or "#" above bars is assigned as follows: # or ∗ at $p < 0.05$.
